# Supplementary material for: Bacterial Metabolic Potential in Response to Climate Warming Alters the Decomposition Process of Aquatic Plant Litter—In Shallow Lake Mesocosms
Source: Microorganisms. 2022 Jun 30;10(7):1327. doi: 10.3390/microorganisms10071327 (PMC9316218; doi:10.3390/microorganisms10071327)
Supplement: Supplementary file 1 [file microorganisms-10-01327-s001.zip › microorganisms-1772191-supplementary.pdf]

Supplementary Materials

Table S1 The bacterial function in leaf and stem litter related to the carbon cycle

Functions	Mean±SE (leaf)	Mean±SE (stem)
Chemo-1	8548±2011	9614±2560
Methylotrophy	1560±367	1265±291
Photoheterotrophy	1707±312	1912±386
Oxygenic photoautotrophy	1200±403	1310±370
Fermentation	1561±397	1772±199
Chemo-2	1247±405	1904±673
Aromatic compound degradation	189±65	395±183
Anoxygenic photoautotrophy	28±21	16±7
Reductive acetogenesis	31±21	17±7
Aliphatic non methane hydrocarbon degradation	17±7	20±6

Table S2 The bacterial function in leaf and stem litter related to the nitrogen cycle

Functions	Mean±SE (leaf)	Mean±SE (stem)
Nitrogen fixation	857±230	771±225
Ureolysis	659±148	1151±313
Nitrate reduction	433±113	1199±690
Ammonification	84±48	633±596
Denitrification	3±2	4±1
Nitrification	0±0	0±0

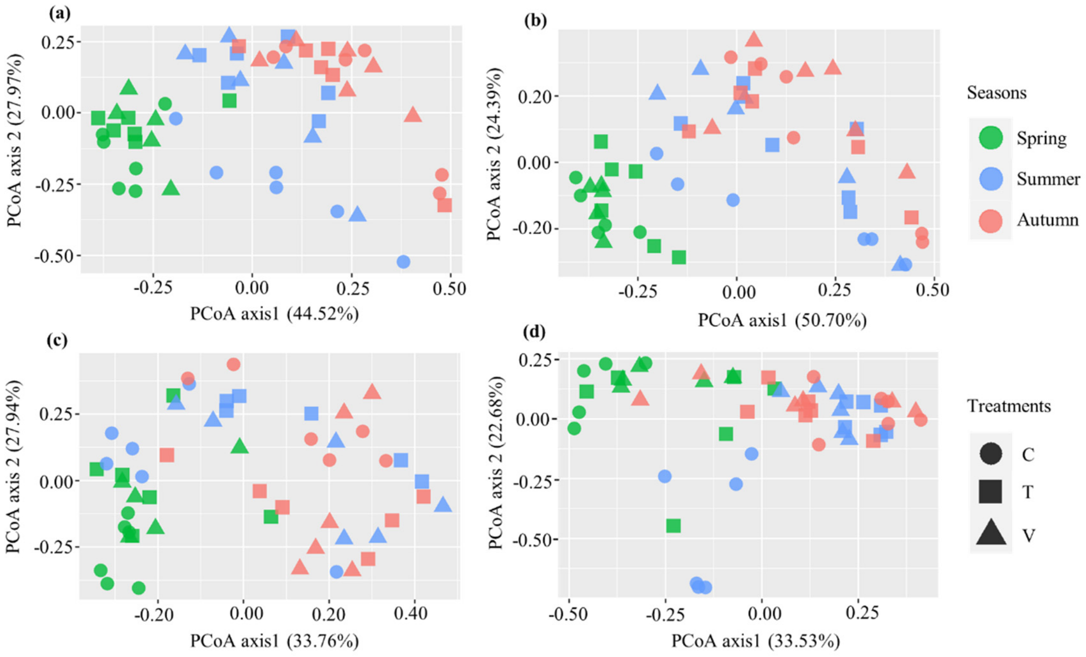

Figure S1. Plots for principal coordinates analysis (PCoA) based on the Bray-Curtis dissimilarities of bacterial function related to carbon cycle in leaf (a) and stem (b) litter, and Plots for principal coordinates analysis (PCoA) based on the Bray-Curtis dissimilarities of bacterial function related to nitrogen cycle in leaf (c) and stem (d) litter.

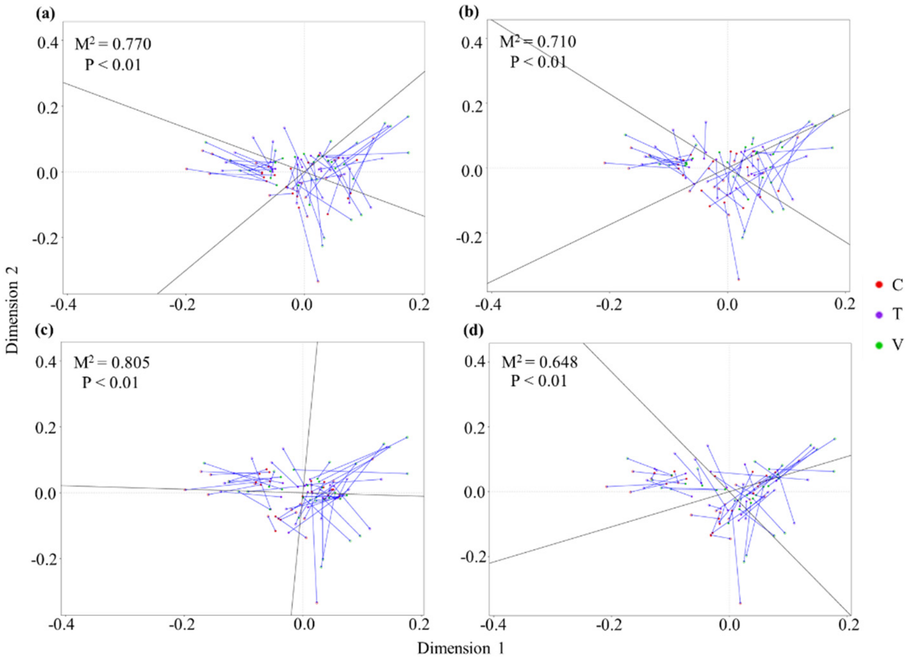

Figure S2. Procrustes analysis of bacterial function related to carbon cycle in leaf (a) and stem (b) litter,

and procrustes analysis of bacterial function related to nitrogen cycle in leaf (c) and stem (d) litter. The points mapped on the main orthogonal axis are quadrats from the environmental variable PCA, and the points mapped on the oblique orthogonal axis are quadrats from the bacterial functional community constituting the PCA. The arrows indicate the paired quadrats from the two. The smaller M^2 is, the higher the correlation degree of the two data sets is, and P reaches the significance level ($p < 0.05$), that the two data sets showed greater consistency.

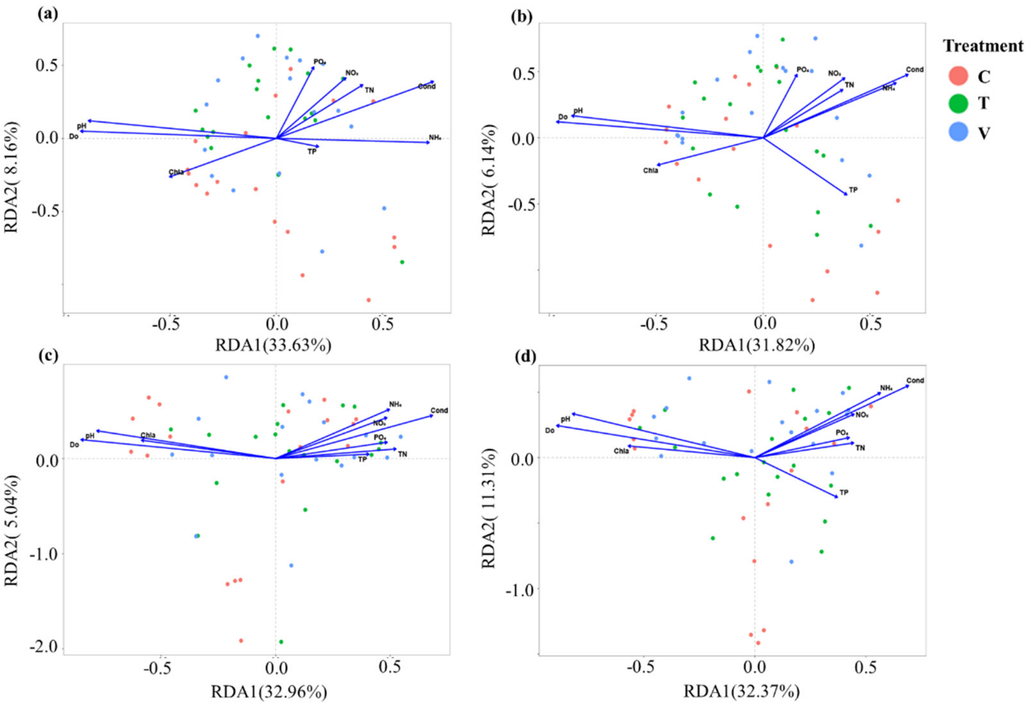

Figure S3. Redundancy analysis of bacterial function and environmental factors related to the carbon cycle of leaf (a) and stem (b) litter and the nitrogen cycle of leaf (c) and stem (d) litter.
